# Heme-Induced Macrophage Phenotype Switching and Impaired Endogenous Opioid Homeostasis Correlate with Chronic Widespread Pain in HIV

**DOI:** 10.3390/cells12121565

**Published:** 2023-06-06

**Authors:** Tanima Chatterjee, Itika Arora, Lilly B. Underwood, Terry L. Lewis, Juan Xavier Masjoan Juncos, Sonya L. Heath, Burel R. Goodin, Saurabh Aggarwal

**Affiliations:** 1 Department of Anesthesiology and Perioperative Medicine, Division of Molecular and Translational Biomedicine, PBMR 230, 901 19th Street South, Birmingham, AL 35205, USA; tchatterjee@uabmc.edu (T.C.); lillyunderwood@uabmc.edu (L.B.U.); terrylewis@uabmc.edu (T.L.L.); jxjuncos@uabmc.edu (J.X.M.J.); 2 Division of Developmental Biology and the Reproductive Sciences Center, Cincinnati Children’s Hospital, Cincinnati, OH 45229, USA; Itika.Arora@cchmc.org; 3 Division of Infectious Disease, University of Alabama at Birmingham, Birmingham, AL 35205, USA; slheath@uabmc.edu; 4 Washington University Pain Center, Department of Anesthesiology, Washington University in St. Louis, St. Louis, MO 63130, USA; burel@wustl.edu

**Keywords:** HIV, chronic widespread pain, cell-free heme, macrophage phenotype, β-endorphin, toll-like receptor-4

## Abstract

Chronic widespread pain (CWP) is associated with a high rate of disability and decreased quality of life in people with HIV-1 (PWH). We previously showed that PWH with CWP have increased hemolysis and elevated plasma levels of cell-free heme, which correlate with low endogenous opioid levels in leukocytes. Further, we demonstrated that cell-free heme impairs β-endorphin synthesis/release from leukocytes. However, the cellular mechanisms by which heme dampens β-endorphin production are inconclusive. The current hypothesis is that heme-dependent TLR4 activation and macrophage polarization to the M1 phenotype mediate this phenomenon. Our novel findings showed that PWH with CWP have elevated M1-specific macrophage chemokines (ENA-78, GRO-α, and IP-10) in plasma. In vitro, hemin-induced polarization of M0 and M2 macrophages to the M1 phenotype with low β-endorphins was mitigated by treating cells with the TLR4 inhibitor, TAK-242. Similarly, in vivo phenylhydrazine hydrochloride (PHZ), an inducer of hemolysis, injected into C57Bl/6 mice increased the M1/M2 cell ratio and reduced β-endorphin levels. However, treating these animals with the heme-scavenging protein hemopexin (Hx) or TAK-242 reduced the M1/M2 ratio and increased β-endorphins. Furthermore, Hx attenuated heme-induced mechanical, heat, and cold hypersensitivity, while TAK-242 abrogated hypersensitivity to mechanical and heat stimuli. Overall, these results suggest that heme-mediated TLR4 activation and M1 polarization of macrophages correlate with impaired endogenous opioid homeostasis and hypersensitivity in people with HIV.

## 1. Introduction

People with HIV (PWH) have an amplified likelihood of developing chronic widespread pain (CWP), which has been shown to increase the rate of disability and decrease quality of life despite having a low viral load and adequate CD4 cell count [1,2,3]. The effectiveness of anti-retroviral therapy (ART) in HIV has been established; however, potential comorbidities such as CWP still impair the daily activities of those with HIV. The prevalence of CWP in PWH ranges between 25% and 85%, and current management is not sufficient to alleviate pain in PWH [1,2,4]. CWP can result from HIV-induced peripheral neuropathy and non-neuropathic inflammation [5,6,7,8]. However, the specific mechanisms that increase the prevalence of CWP in HIV are not well understood. 

The role of macrophages in innate immunity has been well described. Following activation and differentiation due to various stimuli, macrophages develop either pro- or anti-inflammatory properties. Pro-inflammatory macrophages, categorized as M1-like macrophages, produce chemokines and cytokines, leading to an increased inflammatory response. Conversely, anti-inflammatory macrophages, categorized as M2-like macrophages, secrete cytokines that decrease the inflammatory response as well as growth factors that contribute to tissue repair [9,10]. In conjunction with their pro- and anti-inflammatory properties, macrophages produce endogenous opioid peptides. These peptides bind peripheral opioid receptors, leading to inhibition of nociceptive pain transmission [11,12,13,14]. In addition to their anti-inflammatory properties, M2-like macrophages release greater concentrations of these endogenous opioid peptides compared to M1-like macrophages, further reducing pain associated with the inflammatory response [15]. 

People with HIV accompanied by CWP exhibit enhanced hemolysis, elevated plasma levels of heme, and low β-endorphin levels [16]. While essential for various physiological functions, heme, once liberated from red blood cells (RBCs), is deleterious to the body and has been associated with cell damage and the release of pro-inflammatory factors [17,18,19]. Heme molecules have a high affinity for TLR4 receptors on immune cells. Heme activates a proinflammatory response by binding and activating toll-like receptor 4 (TLR4) [20,21], thereby inducing the release of pro-inflammatory cytokines (e.g., IL-1α, IL-6, TNF-α) and chemokines (e.g., IP-10, ENA-78, GRO-α) [18,22,23] that promote hyperalgesia. These inflammatory factors can bind directly to nociceptors and induce neuroinflammation [24]. The expression of various TLRs (e.g., TLR2 and TLR4) on the surface of macrophages regulates the synthesis of pro-inflammatory cytokines and chemokines [25]. Specifically, the pro-inflammatory cascade is activated by TLR4 and is critical for pain induction and, more importantly, leading to the transition from acute to chronic pain [26,27]. 

The objective of the present study was to examine the role of cell-free heme in macrophage polarization and to investigate whether TLR4 activation is involved in heme-induced M1 polarization and impaired endogenous opioid synthesis and release in macrophages. Thus, in the current study, we hypothesized that heme-dependent TLR4 activation polarizes M0 macrophages to the M1 phenotype, which contains lower amounts of β-endorphin than M2 phenotype macrophages. 

## 2. Methods

### 2.1. Human Participants and Measurements

Participant categories were: (1) HIV-negative, pain-negative, and without chronic disease, which may contribute to and be a confounding factor if hemolysis is present; (2) HIV-negative, pain-positive (low back pain); (3) HIV-positive, pain-negative; and (4) HIV-positive, CWP-positive. Category 1 and 2 participants were recruited from UAB as well as the surrounding communities via flyers and word of mouth. Low back pain was the primary pain associated with PWH and CWP; therefore, inclusion criteria for Category 2 participants required that they had experienced chronic low back pain for three consecutive months and/or a minimum of half the days during the previous six-month period [16]. Participants who had undergone any form of low back surgery or had experienced significant trauma/accident within the past year were not recruited. Category 3 and 4 participants were identified from the UAB Center for AIDS Research Network of Integrated Clinical Systems (CNICS) site. Specifically, a review of pain-specific patient-reported outcomes (PROs), which included a brief chronic pain questionnaire where patients reported intensity and duration of pain, identified individuals for recruitment for the present study [8]. The majority of HIV-positive participants at the UAB HIV clinic were on intregrase inhibitor (bictegravir, dolutegravir)-based regimens paired with two nucleoside reverse transcriptase inhibitors (NRTI) (tenofovir, alfenomide). A smaller portion were on non-nucleoside reverse transcriptase inhibitor (NNRTI) (doravirine, rilpivirine)-based regimens paired with 2-NRTIs. Patients were on these therapies for at least 5 years, and there were no significant differences with regard to anti-retroviral therapies between people with HIV with and without chronic pain.

Following collection from participants, blood samples were centrifuged at 2500 g for 10 min at 25 °C in order to separate RBCs from plasma. The plasma-containing supernatant was carefully removed and transferred to a separate tube, then stored at −80 °C until further analysis. Freeze-thaw cycles were avoided to prevent degradation of the samples. Human IP-10, ENA-78, and GRO-α were measured in plasma using U-PLEX Custom Biomarker Assays from Meso scale Discovery (catalog no. K15067M-1; MSD, Rockville, MD, USA).

### 2.2. Reagents

RPMI-1640 medium (product no. 30-2001), fetal bovine serum (product no. 30-2020), dimethylsulfoxide (product no. 4-X), and penicillin/streptomycin (100 U/mL) (product no. 30-2300) were purchased from the American Type Culture Collection (ATCC) (Manassas, VA, USA). Hemopexin (Hx) (heme scavenger; product no. 16-16-080513) was obtained from Athens Research and Technology (Athens, GA). Hemin (ferric chloride heme; product no. H9039), phenylhydrazine hydrochloride (PHZ) (for induction of hemolytic anemia; product no. 114715), phorbol-12-myristate-13 acetate (PMA) (product no. P8139), Tak-242 (product no. 614316), IL-13 (product no. SRP3274), LPS (product no. L4391), IFN-ϒ (product no. SRP3058), IL-4 (product no. SRP3093), DNase I (product no. 10104159001), Collagenase D (product no. 11088866001), and Dispase II (product no. D4693) were obtained from Sigma-Aldrich (St. Louis, MO, USA). RBC Lysis Buffer (10×) (product no. 420302) and Cell Staining Buffer (product no. 420201) were obtained from BioLegend (San Diego, CA, USA). 

### 2.3. Cell Culture

THP-1 cells (catalog no. TIB-202) were purchased from ATCC and cultured in RPMI-1640 medium, which was supplemented with 10% FBS, 2 mM L-glutamine, 100 IU/mL penicillin, and 100 µg/mL streptomycin. Cells were incubated at 37 °C with 95% humidity and 5% CO_2_.

### 2.4. Macrophage Polarization

For M0 differentiation, THP-1 monocytes were treated with PMA in RPMI media (10 ng/mL, 24 h). The media was then changed, and cells were allowed to incubate for an additional 48 h in RPMI medium. For M1 polarization, THP-1 cells were stimulated with 5 ng/mL PMA for 24 h, followed by incubation in RPMI medium for 24 h. Cells were then stimulated with LPS (10 ng/mL) and IFN-γ (20 ng/mL) for 24 h and then incubated in RPMI-1640 media alone for an additional 24 h [28]. For M2 polarization, THP-1 cells were stimulated with 5 ng/mL PMA for 24 h and then allowed to incubate in RPMI-1640 media alone for 72 h. They were further stimulated with IL-4 (20 ng/mL) and IL-13 (20 ng/mL) for an additional 48 h [29].

### 2.5. Flow Cytometry of THP-1 Cells

THP-1 cells were differentiated into M0 cells and then treated with DMSO (vehicle), hemin (1–40 µM, 24 h), or hemin (25 µM, 24 h) in conjunction with the TLR4 inhibitor, Tak-242 (400 nM, 24 h). At the end of the treatment period, macrophages were trypsinized and incubated with 1 mL of FACS buffer for 15 min at 37 °C. The cell suspension was transferred into a microcentrifuge tube and centrifuged at 2000 rpm for 5 min. Macrophages (about 1 × 10^6^ cells/tube) were then washed twice and incubated on ice with Fc-γ receptor blocking solution for 15 min and stained with fluorochrome-conjugated anti-human antibodies, CD11b (BV605), CD80 (APC), CD86 (BV711), CD163 (BV421), and CD206 (APC-Cy7), in combination with viability dye 7AAD from BioLegend, California, USA, for 30 min at 4 °C in the dark, followed by washing (twice) with the FACS staining buffer (List of antibodies is provided in Supplementary Table S1). Flow cytometry data were acquired using the BD LSRII (Franklin Lakes, NJ, USA) flow cytometer, and the data were analyzed with FlowJo X software (version 18.0).

### 2.6. Mouse Model and Treatments

Adult male and female C57BL/6 mice (20–25 g) were purchased from Jackson Labs (Farmington, CT). All mice were housed under a 12-h light/12 h dark cycle with access to a standard diet and water. At the end of the experiment, mice were euthanized by giving an intraperitoneal injection of ketamine and xylazine to minimize pain and distress. Mice were given an intraperitoneal injection of PHZ (50 mg/kg BW) or saline (vehicle) on two consecutive days. One hour after the second injection, mice were administered a single intra-peritoneal (IP) injection of saline (vehicle), hemopexin (4 mg/kg BW), or Tak-242 (3.0 mg/kg) in 1x phosphate buffered saline (PBS). Mouse blood and tissue samples were collected four days after the second PHZ injection.

### 2.7. Preparation of Single Cell Suspension from Mouse Liver

Liver cells were isolated from mice through a modified version of the enzymatic digestion technique described by Li et al. [30]. Briefly, this was accomplished by perfusing the liver through the portal vein with the liver perfusion medium (1× PBS supplemented with 0.2 mM ethylenediaminetetraacetic acid, 10 mM HEPES, and 5 mM glucose) at a rate of 10 mL/min. Immediately after the perfusion, a cut was made in the inferior vena cava below the liver to release blood and perfusion medium from the liver until it appeared completely clear of color. The liver was then resected from the body cavity. The left medial and lateral lobes were used for histology and western blot, while the right lateral lobe was used for flow cytometry. 

The right lateral lobe of the liver was immediately transferred into a prepared digestion buffer after fine mincing. For single-cell suspension, the liver mixture was placed on a shaker (37 °C for 30 min) to ensure complete digestion occurred. Subsequently, cell suspension was passed through a pre-hydrated 70 µm cell strainer placed onto a 50-mL falcon tube with 5 mL of cold complete DMEM media. Dissociated liver cells were centrifuged at 50 G for 3 min at 4 °C and then at 100 G for 2 additional minutes to separate hepatocytic (pellet) from non-hepatocytic cells (supernatant). The supernatant was carefully decanted into a new tube and centrifuged at 165 G for 7 min at 4 °C, and the pellet was resuspended in cold 1× red blood cell lysis buffer. Additionally, non-parenchymal cells were centrifuged at 160 G for 5 min and resuspended in 1 mL of FACS staining buffer. Cell viability was determined by staining with trypan blue and counting live cells using a hemocytometer. Hepatic macrophage-enriched fractions were then stained for flow cytometry analysis or kept frozen for mRNA analysis.

### 2.8. Flow Cytometric Analysis of Single Cell Macrophage Suspension from Liver

Hepatic macrophages were resuspended in FACS staining buffer and incubated on ice with Fc-γ receptor blocking solution for 15 min. Cells were washed by adding 800 µL of FACS buffer and centrifuged. Pellet was then incubated for 45 min at 4 °C with the following antibodies: anti-mouse CD11b (Alexa Fluor 488), F4/80 (APC), CD80 (BV510), CD86 (PE), CD206 (APC-Cy7), and CD163 (BV421), in combination with the viability dye 7AAD from BioLegend, San Diego, CA, USA (a list of antibodies is provided in Supplementary Table S1). Cells were also incubated with a control isotype corresponding to each primary antibody. Cells were then washed and passed through a 70 µm cell strainer into a 5-mL polystyrene round-bottom flow tube. Data were acquired by a FACS Attune Flow Cytometer (BD Biosciences, Bergen County, NJ, USA) and analyzed using FlowJo software.

### 2.9. cDNA Isolation and Real-Time PCR

Total RNA was isolated from THP-1 cells and mice’s livers using Trizol (TRI Reagent by Sigma Life Science) and purified using the RNAeasy Mini Spin Column in the AllPrep DNA/RNA Mini Kit (product no. 80204, QIAGEN, Hilden, Germany). RNA purity and concentration were determined using NanoDrop (Thermo Scientific, Waltham, MA, USA). One microgram of total RNA was reverse transcribed from RNA using a cDNA Synthesis Kit (product number 18080-051, Invitrogen, Thermo Fisher Scientific) and the T100 Thermal Cycler (Bio-Rad, Contra Costa County, CA, USA). cDNAs were amplified using PowerSYBR Green PCR Master Mix (Product number 4367659, Applied Biosystems by Thermo Scientific) in the CFX Opus 96 Real-time PCR System (Bio-Rad). Primers and PCR conditions employed to study gene expression are in Supplementary Table S2. β-actin was considered a housekeeping gene. Relative fold change was measured from the ct value using ∆∆ct method [31,32].

### 2.10. Heme Quantification

Mouse plasma heme concentration was measured using the QuantiChrom heme assay kit (product no. DIHM-250; BioAssay Systems, Hayward, CA, USA), according to manufacturer’s instructions. 

### 2.11. β-Endorphin Assay

Cellular β-endorphin levels were measured using QuickDetect beta-endorphin ELISA kits according to the manufacturer’s instructions (human: product no. E4458-100; mouse: product no. E4459-100; BioVision, Milpitas, CA, USA). 

### 2.12. Behavioral Assay

To assess pain sensitivities to different stimuli, mice were placed in their respective enclosures (Bioseb, Pinellas Park, USA; product no. BIO-VF-M) and allowed to acclimate for at least 1 h prior to performing mechanical (von Frey), cold (acetone evaporation test), and thermal (hot plate) test sessions.

### 2.13. Von Frey Microfilament Test

To measure mechanical sensitivity, we placed mice on a mesh floor inside a plexiglass chamber for 1 h to allow for acclimatization prior to testing. Mechanical allodynia was measured by the simplified up-down method [33]. Briefly, calibrated monofilaments (Aesthesio, Bioseb, Pinellas Park, FL, USA; product no. BIO-PVF) were applied to the glabrous skin of each hind paw to determine the paw withdrawal threshold. Filaments numbered 1.65 through 4.31 were used, and tests were always started with filament 3.22. If no withdrawal was apparent for a given stimulus, the next higher-numbered monofilament was applied; if withdrawal occurred, the next lowest monofilament was applied. Five responses were recorded per hind paw. An average of the final responses from both paws was depicted as the final withdrawal score. The filament values were converted to force (grams), and the data are expressed in percent change from baseline (pretreatment).

### 2.14. Acetone Evaporation Test

To test thermal (cold) allodynia in mice, room-temperature acetone was sprayed onto the center of the mouse’s hind paw using a syringe. Mice were then observed for 60 s and scored based on the number of reactions. Positive reactions included flicking or licking of the tested paw. If the mouse had no reaction, the score was 0; if the mouse had only one reaction, the score was 1; if the mouse had two or more reactions, the score was 2. The test was then repeated on the other paw, and the average of the scores from both paws was taken. Prior to applying acetone, room-temperature water was sprayed on each paw as a control.

### 2.15. Hot Plate Test

Thermal hyperalgesia was determined in mice using a specifically designed hot plate (MazeEngineers, Skokie, IL, USA). The plate temperature was set to 55 °C. Mouse was placed on the hot plate, and a timer was started. Once a response was observed, the timer was stopped and the mouse was removed. Responses included licking or flicking of the back paws or jumping off the plate. If no response was observed within 30 s, the mouse was removed. The latency to response was measured in seconds and reported.

### 2.16. Statistics

Statistical analysis was performed using GraphPad Prism version 9.5 for Windows (GraphPad Software, San Diego, CA, USA). Results were expressed as the mean ± standard error of the mean (SEM). Statistical significance was determined by an unpaired *t*-test for two groups or a one-way ANOVA followed by Tukey’s post-hoc test for more than two groups; *p* < 0.05 was considered significant.

### 2.17. Study Approval

This study was conducted in accordance with approved human subject protocols by the University of Alabama at Birmingham (UAB) Institutional Review Board (IRB Protocols 300000107 and 170119003). All animal care and experimental procedures were approved by the Institutional Animal Care and Use Committee at the UAB (Animal Protocol number: 22344).

## 3. Results

### 3.1. M1 Macrophage-Specific Chemokines Were Elevated in PWH with CWP

The aim of the study was to determine whether elevated levels of M1 macrophages, which are deficient in endogenous opioids, are responsible for HIV-associated pain. Human participants were recruited for the study and divided into four groups: (1) HIV-negative, pain-negative, and without chronic disease; (2) HIV-negative, pain-positive (low back pain); (3) HIV-positive, pain-negative; and (4) HIV-positive and CWP-positive, as published earlier [16]. Demographic and clinical information were recorded for each individual recruited for this study. Participants in all groups were between the ages of 45 and 54 years and were predominantly African American (64–77%). The proportion of men in the HIV-positive groups was higher than in the HIV-negative groups, which reflects the patient population of the HIV clinic at UAB. The average current and nadir CD4^+^ cell counts and the average current and highest viral load (VL) were not different between HIV-positive individuals with or without CWP. Mean plasma heme levels in PWH were 80.9 µM vs. 14.4 µM for the HIV-negative without pain group, 26.3 µM for the HIV-negative with pain group, and 27.4 µM for the HIV-positive without pain group. The viral load in all HIV-1-positive study participants was insignificant as they were on antiretroviral therapy (Table 1).

**Figure 1:**
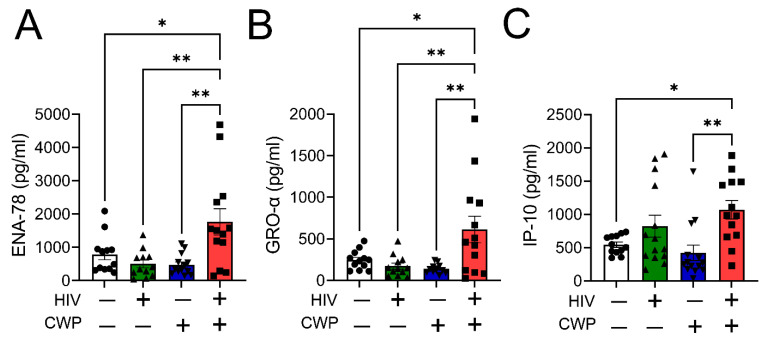
People with HIV with CWP have increased plasma levels of M1 macrophage-specific chemokines. HIV-1 positive individuals with CWP exhibited significantly higher plasma levels of M1-specific chemokines, ENA-78 (*n* = 14) (**A**), GRO-α (*n* = 14) (**B**), and IP-10 (*n* = 14) (**C**), compared to HIV-1 negative individuals with or without pain and HIV-1 positive people without pain. Individual values and means ± SEM. * *p* < 0.05, ** *p* < 0.01 vs. groups at the end of individual lines; one-way ANOVA followed by Tukey post-hoc testing.

In our previous study, PWH who self-reported CWP had elevated plasma levels of pro-inflammatory and pro-algesic cytokines such as IL-1β, IL-6, and TNF-α compared to HIV-negative individuals with or without pain [16]. In the current experiments, the plasma profiles of three chemokines, which are exclusively expressed and released by M1 macrophages, were quantified in participants. Our data showed that PWH with CWP exhibit significantly higher levels of chemokines, ENA-78 (Figure 1A) and GRO-α (Figure 1B), compared to other study participants who were HIV-1 positive without pain or HIV-1 negative with or without pain. Plasma IP-10 (Figure 1C) levels were also elevated in PWH with CWP in comparison to the HIV-1-negative participants. These markers are produced predominantly by pro-inflammatory M1 rather than pro-resolution M2 macrophages [23]. 

### 3.2. Heme-Induced Transition of M0 and M2 Macrophages to M1 Phenotype

**Figure 2:**
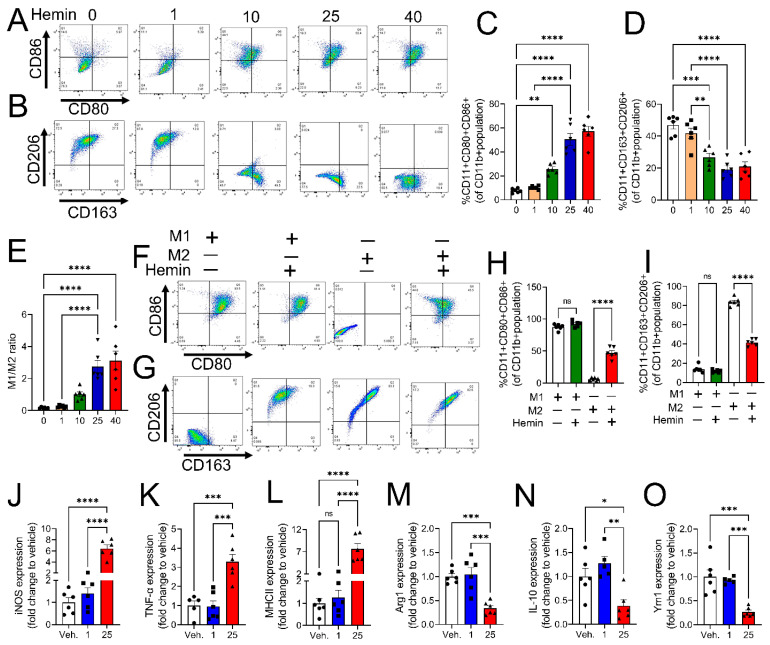
Hemin-induced transition of M0 and M2 macrophages to M1 phenotype. Monocyte-derived THP-1-M0 macrophages were treated with varying concentrations of hemin for 24 h, and the expression of M1 and M2 macrophage polarization markers was measured using flow cytometry. Histogram (**A**) and bar graph (**C**) demonstrate that hemin increased the expression of M1 polarization markers, CD80 and CD86 (*n* = 6) and decreased the expression of M2 polarization markers, CD206 and CD163 (*n* = 6) (**B**,**D**). There was a significant increase in the M1/M2 population with increasing concentrations of hemin (*n* = 6) (**E**). Next, fully differentiated M1 or M2 cells were challenged with hemin (25 µM) for 24 h. Flow cytometry histograms and bar graphs showed that hemin treatment increased the transition of M2 cells towards the M1 phenotype (*n* = 6) (**F**,**H**). In addition, hemin significantly reduced the M2 population (*n* = 6) (**G**,**I**). RT-PCR was performed using total RNA extractions from PMA-stimulated THP-1-M0 macrophages treated with hemin. Gene expression studies revealed a significant increase in M1-associated markers like iNOS, TNFα, and MHCII (*n* = 6) (**J**–**L**) and a decrease in M2 markers, Arg-1, IL-10, and Ym1 (*n* = 6) (**M**–**O**) when compared with (DMSO) vehicle-treated M0 macrophages. Individual values and means ± SEM. * *p* < 0.05, ** *p* < 0.01, *** *p* < 0.001, **** *p* < 0.0001 vs. groups at the end of individual lines; one-way ANOVA followed by Tukey post-hoc testing.

We have previously shown that PWH with CWP has increased hemolysis and high plasma levels of cell-free heme [23]. Therefore, in this study, we determined whether cell-free heme is responsible for macrophage polarization to the M1 phenotype. PMA-stimulated THP-1-MO macrophages were treated with varying concentrations of hemin (ferric chloride heme; 1 µM–40 µM) for 24 h, and the expression of M1 and M2 macrophage polarization markers was measured using flow cytometry (the gating strategy is shown in Supplementary Figure S1). Hemin increased the expression of M1 polarization markers, CD80 and CD86 (Figure 2A,C) and decreased the expression of M2 polarization markers, CD206 and CD163 (Figure 2B,D). This response was dependent on the concentration of hemin. The increase in M1 and the decline in M2 phenotype populations were noted at 10µM and peaked at 25 µM (Figure 2A–D). To characterize the efficacy of M1/M2, polarization dot plots were used to identify M1 cells that were double-positive for CD80 and CD86 (CD80+ CD86+) and M2 cells that were double-positive for CD163 and CD206 (CD163+ CD206+). Results showed a significant increase in the M1/M2 population with increasing concentrations of hemin (Figure 2E). Next, monocyte-derived THP-1 cells were stimulated with cytokines to polarize them to the M1 or M2 macrophage phenotype. Fully differentiated M1 or M2 cells were then challenged with hemin (25 µM) for 24 h. Flow cytometry results showed that hemin treatment increased the transition of M2 cells towards the M1 phenotype (Figure 2F,H). In addition, hemin significantly reduced the M2 population (Figure 2G,I). To further validate these results, RT-PCR was performed using total RNA extractions from PMA-stimulated THP-1-M0 macrophages treated with hemin. Gene expression studies showed a significant increase in M1-associated markers, like iNOS, TNFα, and MHCII (Figure 2J–L), as well as a decrease in mRNA levels of M2 markers, Arg-1, IL-10, and Ym1, in hemin-exposed M0 cells compared to vehicle-treated cells (Figure 2M–O). Overall, these results showed that heme induces the polarization of macrophages toward an M1-like proinflammatory phenotype.

### 3.3. Heme-Induced M1 Phenotype Transition Is Mediated by TLR4

**Figure 3:**
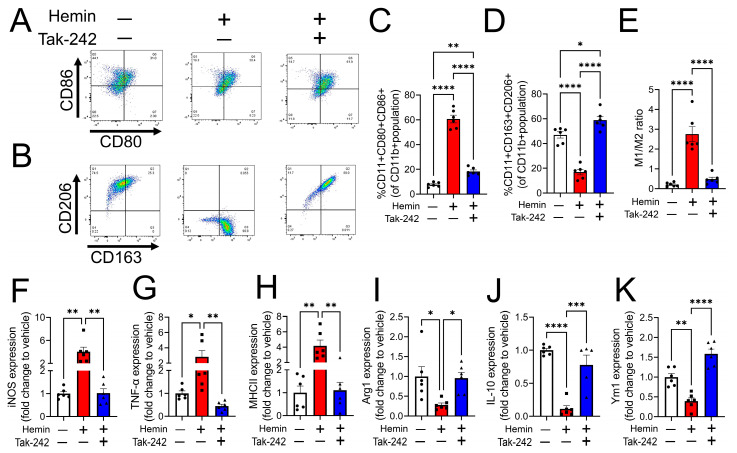
Heme-induced M1 phenotype transition is mediated by TLR4. PMA-stimulated THP-1-M0 macrophages were treated either with DMSO (vehicle), hemin (25 µM), or hemin along with Tak-242 (400 nM) for 24 h, and the expression of M1 and M2 macrophage polarization markers were measured. A flow cytometry histogram and bar graph demonstrated that Tak-242 attenuated the hemin-induced increase in the expression of M1 polarization markers CD80 and CD86 (*n* = 6) (**A**,**C**) and the decrease in the expression of M2 polarization markers CD206 and CD163 (*n* = 6) (**B**,**D**). Tak-242 also decreased the M1/M2 ratio in hemin-challenged cells (*n* = 6) (**E**). Gene expression studies using RT-PCR showed that treatment of M0 macrophages with Tak-242 attenuated a heme-dependent increase in M1 markers, iNOS, TNFα, and MHCII (*n* = 6) (**F**–**H**) and a decrease in M2 markers, Arg-1, IL-10, and Ym1 (*n* = 6) (**I**–**K**). Individual values and means ± SEM. * *p* < 0.05, ** *p* < 0.01, *** *p* < 0.001, **** *p* < 0.0001 vs. groups at the end of individual lines; one-way ANOVA followed by Tukey post-hoc testing.

Heme is known to act as a proinflammatory molecule by binding and activating toll-like receptor 4 (TLR4), thus inducing the production of inflammatory cytokines in leukocytes [20,21]. Here, we investigated whether TLR4 activation is involved in heme-induced M1 polarization of macrophages. PMA-stimulated THP-1-M0 macrophages were treated either with DMSO (vehicle), hemin (25 µM), or hemin along with the TLR4 inhibitor, Tak-242 (400 nM) for 24 h, and expression of M1 and M2 macrophage polarization markers was measured using flow cytometry. Tak-242 attenuated the hemin-induced increase in the expression of M1 polarization markers CD80 and CD86 (Figure 3A,C) and the decrease in the expression of M2 polarization markers CD206 and CD163 (Figure 3B,D). Further analysis of flow cytometric data revealed a decrease in the M1/M2 ratio when hemin-challenged cells were treated with Tak-242 (Figure 3E). Finally, gene expression studies using RT-PCR showed that treatment of M0 macrophages with Tak-242 attenuated a heme-dependent increase in M1 markers, iNOS, TNFα, and MHCII (Figure 3F–H), and a decrease in M2 markers, Arg-1, IL-10, and Ym1 (Figure 3I–K). These results suggested that heme-induced polarization of macrophages toward a M1-like proinflammatory phenotype is mediated by TLR4.

### 3.4. Heme Scavenging or TLR4 Inhibition Attenuated Monocyte/Macrophage Phenotype Transition in a Mouse Model of Hemolysis

**Figure 4:**
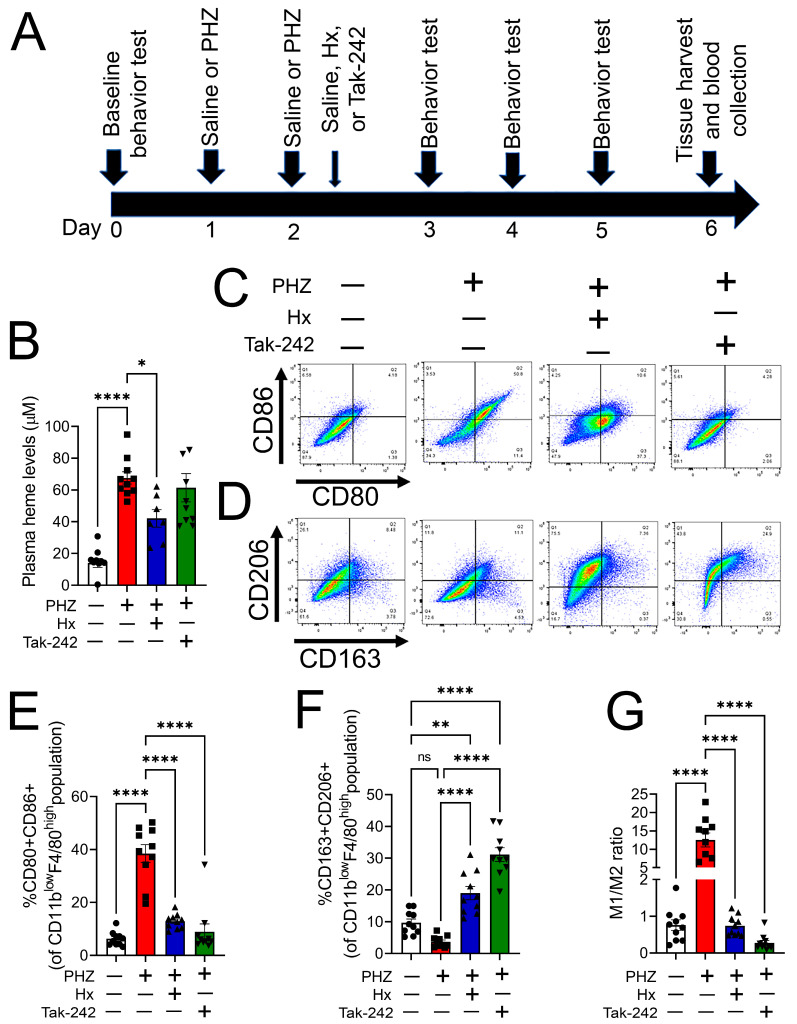
Heme scavenging or TLR4 inhibition attenuated macrophage phenotype transition in a mouse model of hemolysis**.** Phenylhydrazine hydrochloride (PHZ, 50 mg/kg, IP) or saline was administered to adult male and female C57BL/6 mice on two consecutive days. One hour following the 2nd PHZ injection, a subset of mice was given saline (vehicle, IP), purified human hemopexin (Hx) (4 mg/kg, IP) or Tak-242 (3.0 mg/kg, IP) (**A**). Four days post-second PHZ injection, Hx but not Tak-242 attenuated the PHZ-induced increase in plasma heme levels (**B**). A flow cytometry histogram (**C**) and bar graph (**E**) showed an increase in the expression of M1 markers CD80 and CD86 in hepatic macrophages of PHZ-treated animals, while the expression of these markers was not increased in mice that received either Hx or Tak-242 along with PHZ [*n* = 10 (5 males and 5 females)]. M2 markers, CD206 and CD163, showed a significant increase in Hx and Tak-242-treated mice but no significant decrease in the PHZ-treated mice [*n* = 10 (5 males and 5 females)] (**D**,**F**). The ratio of M1/M2 hepatic macrophage population showed a significant increase in PHZ-treated mice but not in mice that received either Hx or Tak-242 post-PHZ challenge [*n* = 10 (5 males and 5 females)] (**G**). Individual values and means ± SEM. * *p* < 0.05, ** *p* < 0.01, *** *p* < 0.001, **** *p* < 0.0001 vs. groups at the end of individual lines; one-way ANOVA followed by Tukey post-hoc testing.

To assess whether heme-dependent TLR4 activation altered macrophage plasticity in vivo, a bolus injection of PHZ (50 mg/kg, IP) or saline was given to adult male and female C57BL/6 mice on two consecutive days. A subset of mice received saline (vehicle, IP), purified human hemopexin (Hx) (4 mg/kg, IP), or Tak-242 (3.0 mg/kg, IP) 1 h following the 2nd PHZ injection. On day 4 post-second PHZ injection, blood and tissues were harvested (Figure 4A). Four treatment groups of mice (5 males and 5 females) were used in this study: saline (vehicle); PHZ; PHZ + Hx; PHZ + Tak-242. Hx but not Tak-242 treatment significantly reduced plasma levels of cell-free heme in PHZ-challenged mice (Figure 4B). Markers of M1 and M2 polarization were analyzed by flow cytometry in liver macrophages (gating strategy shown in Supplementary Figure S2). Hepatic macrophages showed an increase in the expression of M1 markers CD80 and CD86 in PHZ-treated animals. In contrast, the expression of these markers was not increased in mice that received either Hx or Tak-242 along with PHZ (Figure 4C,E). Though there was no significant decrease in the expression of M2 markers CD206 and CD163 in PHZ-treated mice, the M2 markers were significantly increased in Hx and Tak-242-treated mice (Figure 4D,F). The ratio of the M1/M2 hepatic macrophage population was significantly increased in PHZ-treated mice but not in mice that received either Hx or Tak-242 post-PHZ challenge (Figure 4G). 

**Figure 5:**
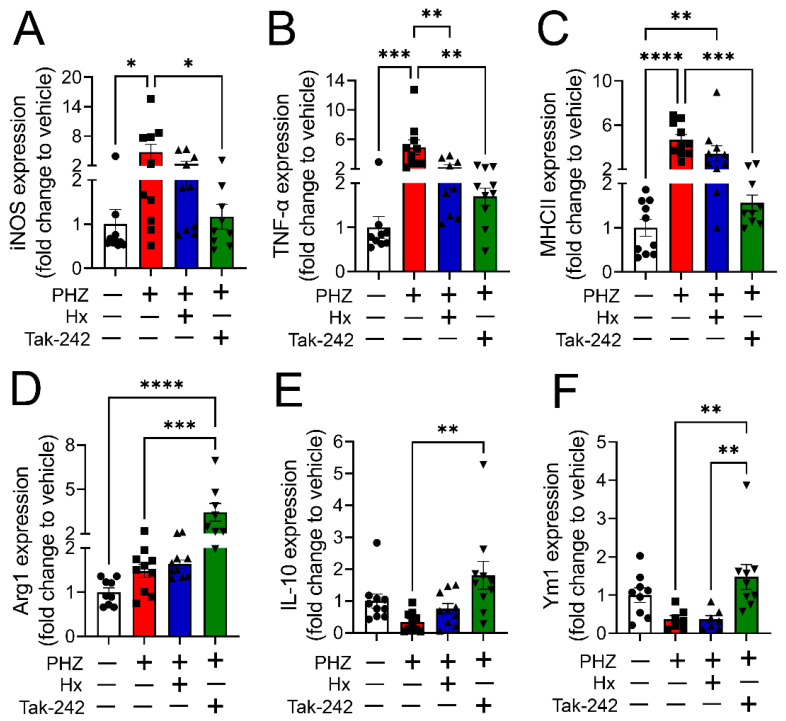
Heme scavenging or TLR4 inhibition altered M1 and M2 markers in a mouse model of hemolysis. The mRNA expression of M1 markers, iNOS, and TNF-α was significantly increased in hepatic macrophages in PHZ-treated mice but not in mice that received either Hx or Tak-242 in conjunction with PHZ [*n* = 10 (5 males and 5 females)] (**A**,**B**). Tak-242 also decreased MHCII mRNA expression in PHZ-challenged mice [*n* = 10 (5 males and 5 females)] (**C**). The mRNA expression of M2 markers, Arg-1, IL-10, and Ym1, was significantly higher in mice that received Tak-242 along with PHZ compared to mice that received only PHZ [*n* = 10 (5 males and 5 females)] (**D**–**F**). Individual values and means ± SEM. * *p* < 0.05, ** *p* < 0.01, *** *p* < 0.001, **** *p* < 0.0001 vs. groups at the end of individual lines; one-way ANOVA followed by Tukey post-hoc testing.

The mRNA expression of M1 markers, iNOS, TNF-α, and MHCII, increased in PHZ-treated mice but not in mice that received either Hx or Tak-242 in conjunction with PHZ (Figure 5A–C). Further, the mRNA expression of M2 markers, Arg-1, IL-10, and Ym1, was significantly higher in mice that received Tak-242 along with PHZ compared to mice that received only PHZ (Figure 5D–F). These results confirmed our in vitro findings in Figure 3 that heme induces polarization of macrophages to the M1 phenotype and is mediated by the activation of TLR4. 

### 3.5. Heme Scavenging or TLR4 Inhibition Ameliorated Impaired Opioid Homeostasis and Hypersensitivity in a Mouse Model of Hemolysis

**Figure 6:**
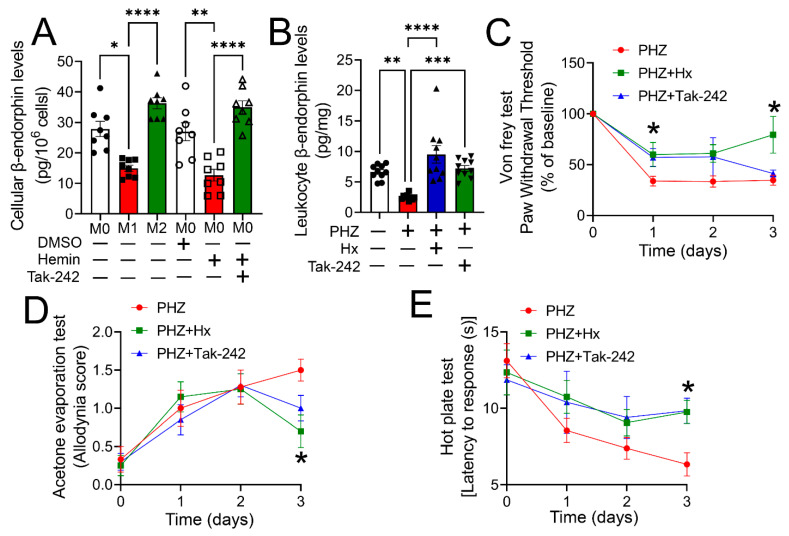
Heme scavenging or TLR4 inhibition ameliorated impaired opioid homeostasis and hyperalgesia in a mouse model of hemolysis**.** β-endorphin levels were measured in cytokine-stimulated M1 and M2 macrophages. M1 macrophages had significantly lower levels of β-endorphin when compared with M2 macrophages and M0 macrophages (*n* = 8) (**A**). Next, THP-1-M0 macrophages were exposed to DMSO (vehicle), hemin (25 µM), or hemin together with Tak-242 (400 nM) for 24 h. Tak-242 abrogated the hemin-induced decrease in β-endorphin levels in cells (*n* = 8) (**A**). β-endorphins were measured in the leukocytes of hemolytic mice. Hx or TAK-242 treatment rescued the decline in β-endorphin levels in PHZ-challenged mice [*n* = 10 (5 males and 5 females)] (**B**). Mechanical allodynia analysis by Von Frey testing showed that paw withdrawal threshold was significantly higher 1-day post-PHZ exposure in Tak-242 treated mice and 3-days post-PHZ in Hx treated mice [*n* = 10 (5 males and 5 females)] compared to mice that received saline post-PHZ exposure (**C**). Thermal allodynia testing with acetone evaporation test showed an increase in allodynia score over a 3-day time period post-PHZ. Hx attenuated PHZ-induced changes [*n* = 10 (5 males and 5 females)] (**D**). Thermal hyperalgesia testing by hot plate showed a decrease in latency to response in PHZ-challenged mice that received saline but not in mice that received either Hx or Tak-242 [*n* = 10 (5 males and 5 females)] (**E**). Individual values and means ± SEM. * *p* < 0.05, ** *p* < 0.01, *** *p* < 0.001, **** *p* < 0.0001 vs. groups at the end of individual lines; one-way ANOVA followed by Tukey post-hoc testing.

Previously, we demonstrated that cell-free heme attenuates β-endorphin production and release from macrophages [16]. Scavenging of cell-free heme in a mouse model of hemolysis improved endogenous opioid homeostasis and reduced heme-induced mechanical hypersensitivity [16]. In the present study, we determined whether heme-induced TLR4 activation and macrophage phenotype transition are responsible for impairing β-endorphin homeostasis and hypersensitivity. First, we measured β-endorphin levels in cytokine-stimulated M1 and M2 macrophages. M1 macrophages had significantly lower levels of β-endorphin when compared with M2 macrophages or M0 macrophages (Figure 6A), suggesting that M1 macrophage polarization attenuated β-endorphin synthesis. Next, THP-1-M0 macrophages were exposed to DMSO (vehicle), hemin (25 µM), or hemin together with the TLR4 inhibitor, Tak-242 (400 nM) for 24 h. Data shows a significant reduction in β-endorphin levels in hemin-challenged cells but not in cells that received Tak-242 along with hemin (Figure 6A). The concentration of β-endorphin was further measured in leukocytes isolated from a mouse model of hemolysis (5 male and 5 female C57BL/6 mice). Groups were saline (vehicle), PHZ, PHZ + Hx, and PHZ + Tak-242, as mentioned earlier. Results demonstrated that β-endorphin levels were significantly lower in PHZ-challenged mice but not in mice that received either Hx or Tak-242 along with PHZ (Figure 6B), suggesting that TLR4 mediates heme-induced opioid dysregulation. 

Next, we assessed whether heme-induced TLR4 activation was involved in hypersensitivity to mechanical, heat, or cold stimuli. Mechanical allodynia was tested by measuring the hind paw withdrawal threshold upon stimulation with Von Frey filaments. The acetone evaporation test was used to measure cold allodynia, while the hot plate test was used to evaluate thermal hyperalgesia. First, baseline mechanical, cold, and thermal sensitivities were measured in mice. Then mice received a bolus dose of PHZ (50 mg/kg BW, IP) on two consecutive days. One hour after the second injection, mice were given a single injection of saline (vehicle, IP), hemopexin (4 mg/kg BW, IP), or Tak-242 (3.0 mg/kg, IP). Pain parameters were measured over the next three days. Von Frey testing showed a significant reduction in paw withdrawal threshold (about 60%) compared to baseline over 3 days in PHZ-treated mice that received saline (Figure 6C). However, compared to saline-treated mice, the paw withdrawal threshold was significantly higher 1-day post-PHZ exposure in Tak-242-treated mice and 3-days post-PHZ in Hx-treated mice (Figure 6C). The thermal allodynia score, an indicator of pain sensation upon acetone evaporation, increased over a 3-day time period post-PHZ but was significantly lower in mice that received Hx (Figure 6D). Finally, the latency to response upon being placed on a hot plate, an indicator of thermal hyperalgesia, showed a significant decline in PHZ-challenged mice that received saline but not in mice that received either Hx or Tak-242 (Figure 6E). These results suggest that TLR4 mediates heme-induced mechanical allodynia and thermal hyperalgesia, while heme-induced thermal (cold) allodynia may be mediated by pathways independent of TLR4. Sex-related differences were not seen in these studies.

## 4. Discussion

PWH have a higher prevalence of intravascular hemolysis than HIV-negative individuals. Some of the reported mechanisms of hemolysis in HIV include low red blood cell (RBC) glutathione, an anti-oxidant required to maintain the normal structure of RBCs [34,35,36]. Anti-retroviral therapy or other drugs for HIV-associated infections, such as amphotericin B and co-trimoxazole for cryptococcal meningitis and interferon and ribavirin for hepatitis C, may also increase the risk of hemolysis [37,38,39]. HIV also confers a 15–40-fold higher risk of acquired thrombotic microangiopathy compared with the HIV-negative population, which is associated with increased intravascular hemolysis [40,41,42]. PWH also have a higher prevalence of glucose-6-phosphate dehydrogenase (G6PD) deficiency, which is an important cause of hemolysis [43]. We previously reported that elevated plasma levels of cell-free heme and low levels of endogenous opioids, like β-endorphin, correlated with self-reported CWP in PWH [16]. A previous study published in 1995, although conducted before the era of Highly Active Antiretroviral Therapy (HAART) (1996–1997), also reported low plasma and brain β-endorphin levels in PWH [44]. Additionally, a recent multicenter AIDS cohort study found that PWH had higher plasma levels of soluble CD14, a marker of classical monocytes that are precursors to M1 macrophages [45], which contain lower amounts of opioid peptides than M2 [46]. Despite an association between hemolysis and pain in several clinical populations [47,48], none have addressed potential mechanisms of heme-induced pain. In our current study, we provided a pioneering report detailing mechanistic insight into how heme causes phenotypic transition of macrophages, which results in decreased levels of β-endorphins and increased pain sensitivity. 

Previously, we had found that PWH with CWP have elevated plasma levels of pro-inflammatory cytokines such as IL-1β, IL-6, and TNF-α. Pro-inflammatory M1 macrophages are the primary source of these cytokines associated with pain. In our current study, we found that plasma levels of M1-specific chemokines, such as ENA-78 (CXCL5), GRO-α (CXCL1), and IP-10 (CXCL10), were also elevated in PWH with CWP, suggesting that an abundance of M1 macrophages may underlie the lack of peripheral endogenous opioids in this patient group. Phenotypic diversity is a hallmark of the macrophage lineage, and it includes pro-inflammatory M1 populations and anti-inflammatory M2 populations [49]. Macrophages are an important source of endogenous opioid peptides that inhibit nociceptive transmission by binding to peripheral opioid receptors [50,51]. Macrophages generate and release opioid peptides like enkephalin, dynorphin, and β-endorphin in response to inflammation or injury. Opioid concentration and frequency of release by the M2-polarized, pro-resolution phenotype are increased over those of macrophages that are of the pro-inflammatory, M1 phenotype [15]. Encouraging the polarization of naive macrophages toward the M2 phenotype tends to attenuate postoperative pain and decrease tactile hypersensitivity [52,53]. Pannell et al. showed that adoptive transfer of M2 cells at the site of injury ameliorated mechanical hypersensitivity, which was reversed by the opioid receptor antagonist naloxone methiodide [15]. Our data aligns with these findings by demonstrating that β-endorphin levels in cytokine-induced M2 polarized cells were 1.5-fold higher in comparison to M0 and 4.5-fold higher in comparison to M1 polarized macrophages. 

The M1/M2 model gives useful conceptual insights into addressing macrophage phenotypes at the site of injury [10,54,55]. Polarization from M0 macrophages towards the M1 phenotype using LPS and IFN-ϒ or towards the M2 phenotype using IL-4 are established procedures that were employed in our study [54,56,57]. Our flow cytometric data showed that M1 cells were double-positive for CD80 and CD86 (CD80+ CD86+), and M2 cells were double-positive for CD163 and CD206 (CD163+ CD206+). CD163 and CD206 are major markers for the identification of M2 macrophages [58,59], while the M1 phenotype expresses CD80, CD86, CD64, and CD32 [60,61,62]. However, neither of these markers is exclusively expressed by M1 or M2 cells; expression of these markers highly correlates with their phenotype. This was particularly evident from the M1 stain, where 95% of M1 cells and 32% of M2 cells were CD80+CD86+, whereas 12% of M1 cells and 83% of M2 cells were positive for the M2-markers, CD163+CD206+, which is clearly consistent with the M1/M2 polarization status reported in the literature [63,64]. Our data was further validated by exhibiting increased mRNA levels of pro-inflammatory cytokines (MHCII, TNFα, iNOS, and IL-6) produced by M1 polarized macrophages and high expression of anti-inflammatory cytokines IL-10 and Arg-1 by M2 polarized macrophages. Inflammatory mediators stimulate the classic activation of macrophages to the M1 phenotype, which, in return, releases high levels of pro-inflammatory cytokines including TNF-α, interleukin-6 (IL-6), and IL-1β. In addition, activated M1 macrophages express inducible nitric oxide synthase (iNOS), which, following binding to its substrate L-arginine, produces nitric oxide (NO). Conversely, expression of Arginase 1 (Arg1) by M2 macrophages culminates in the hydrolysis of L-arginine to L-ornithine. 

Macrophages undergo a process of activation and phenotypic transition upon coming into contact with external stimuli, such as products of cellular injury [65]. In this study, we found that exposing macrophages to hemin or animals to the hemolytic agent PHZ polarized naive M0 cells to the M1 phenotype. PHZ induces lipid peroxidation of erythrocyte membranes, leading to intravascular hemolysis and the release of cell-free heme [66]. A similar model using PHZ has been shown previously in our study to induce hemolysis [16]. Antagonizing TLR4 by Tak-242 prevented this phenotypic transition both in vitro and in vivo, suggesting that heme mediates this effect via binding to TLR4. These observations are interesting in the context of hemolytic diseases associated with pain, such as sickle cell disease [20,67], where monocytes exhibit increased production of TNFα and IL-1β [18,67]. In these scenarios, TLR4 activation is critical for pain induction by directly modulating the proinflammatory cascade [20]. Several other studies have also demonstrated the role of hemoglobin, heme, and iron in macrophage polarization [18,65,68]. In a wound healing model, macrophage iron overload correlated with a proinflammatory M1 phenotype, TNFα, and hydroxyl radical production [69]. Another study observed a decrease in M2 and an increase in M1 macrophages in the red pulp of the spleen and synovial tissue of hemophilic mice [70]. Vinci et al. observed in sickle cell mice that the administration of human exogenous hemopexin attenuates the inflammatory phenotype of macrophages [18]. Thus, our findings are in line with recent studies demonstrating the role of heme in macrophage polarization towards a pro-inflammatory phenotype leading to the activation of inflammatory cytokines via TLR-4 stimulation [20,21,22,71].

Earlier, we reported that an increase in hemolysis correlated with a subsequent decrease in β-endorphins and mechanical hypersensitivity [16]. In this study, we showed that this effect of heme is mediated by TLR4. We further demonstrated that blocking TLR4 in our hemolytic model attenuates mechanical allodynia and thermal hyperalgesia. TLR4 is an important pattern recognition receptor, and its activation is most robustly involved in pain induction by directly modulating the proinflammatory cascade [26,27,72,73,74,75]. However, other TLRs like TLR2, 5, and 9 have also been shown to mediate hypersensitivity [75,76,77,78,79]. Specifically, TLR2 plays an integral role in the maintenance of neuropathic pain by activating NF-kB [80]. Recent studies have shown that several of these TLRs, including 2, 4, 7, and 8, may be activated in HIV [81,82]. In our study, we found that inhibiting TLR4 did not reverse cold hypersensitivity, which was otherwise abrogated by heme scavenging by hemopexin. Therefore, it is entirely possible that activation of other TLRs, such as TLR2, by heme [83] may be involved in heme-induced thermal (cold) allodynia, which is independent of TLR4. 

## 5. Conclusions

In conclusion, our present study successfully elucidated that cell-free heme-induced TLR4 activation and polarization of macrophages to the M1 phenotype may be responsible for the decrease in leukocyte β-endorphin and hypersensitivity in PWH. The limitations of the study include the utilization of PMA-differentiated THP-1 cells as a model to study macrophage polarization instead of primary monocyte-derived macrophages, which may limit physiological relevance to some extent. In addition to highlighting the role of heme in TLR4 activation in HIV, other factors such as HIV proteins, Gp120, and Tat are known to activate TLR4 and TLR2 and therefore may also be responsible for CWP in PWH [82,84,85]. Furthermore, it is entirely possible that antiretroviral drugs may also be responsible for TLR4 activation, macrophage polarization, inhibition of β-endorphin levels, or may induce pain through other mechanisms that are not studied here. Nevertheless, strategies to reduce cell-free heme or inhibit TLR4 may still be important therapeutic avenues to reduce pain in this patient population.

## Figures and Tables

**Table 1:** Demographic and clinical data of study participants: The demographic and clinical data describe average age in years, current and nadir CD4^+^ cell count (cells/mm^3^), average current and highest viral load (VL), and plasma heme levels (µM). Values are mean ± SD. ^a^ *p*  <  0.00001 vs. HIV-negative without pain; ^b^ *p*  <  0.001 vs. HIV-negative with pain; ^c^ *p*  <  0.001 vs. HIV-positive without pain; one-way ANOVA followed by Tukey’s post-hoc testing.

Group (Number of Participants)	HIV_neg_, Pain_neg_ (14)	HIV_neg_, Pain_pos_ (14)	HIV_pos_, Pain_neg_ (14)	HIV_pos_, Pain_pos_ (13)
Avg. age (SD)	50.1 (12.6)	45.3 (15.0)	48.6 (8.4)	54.1 (5.8)
Percent Females	64.3	64.3	50	38.5
Percent Afr. Amer.	64.3	64.3	71.4	76.9
Avg. CD4^+^ (SD)			715.4 (421.2)	637.5 (385.0)
Avg. Nadir CD4^+^ (SD)			165.0 (231.5)	204.5 (202.4)
Avg. VL (SD)			19.5 (1.9)	59.2 (130.7)
Avg. Highest VL (SD)			199115 (249762)	428995 (1220712)
Plasma heme (µM) (SD)	14.4 (4.0)	26.3 (13.0)	27.4 (12.4)	80.9 (62.4) ^abc^

Demographic and clinical data of HIV-positive and HIV-negative participants. Age in years; CD4^+^ cells/mm^3^; VL: viral load copies/mL; SD: standard deviation.

## Data Availability

The data presented in this study are available on request from the corresponding author.

## Supplementary Materials

The following supporting information can be downloaded at: https://www.mdpi.com/article/10.3390/cells12121565/s1. Supplementary Figure S1: THP-1 macrophages gating strategy; Supplementary Figure S2: Liver cells gating strategy; Supplementary Table S1: The table shows the source of all antibodies that were used to perform FACS analysis in the study; Supplementary Table S2: The table shows all the primers that were used to perform real time PCR in the study.

## References

[1] Miaskowski C., Penko J.M., Guzman D., Mattson J.E., Bangsberg D.R., Kushel M.B. (2011). Occurrence and characteristics of chronic pain in a community-based cohort of indigent adults living with hiv infection. J. Pain.

[2] Merlin J.S., Westfall A., Raper J.L., Zinski A., Norton W.E., Willig J.H., Gross R., Ritchie C.S., Saag M.S., Mugavero M.J. (2012). Pain, mood, and substance abuse in hiv: Implications for clinic visit utilization, antiretroviral therapy adherence, and virologic failure. Am. J. Ther..

[3] Madden V.J., Parker R., Goodin B.R. (2020). Chronic pain in people with hiv: A common comorbidity and threat to quality of life. Pain Manag..

[4] Merlin J.S., Cen L., Praestgaard A., Turner M., Obando A., Alpert C., Woolston S., Casarett D., Kostman J., Gross R. (2011). Pain and physical and psychological symptoms in ambulatory hiv patients in the current treatment era. J. Pain Symptom Manag..

[5] Robinson-Papp J., Simpson D.M. (2009). Neuromuscular diseases associated with hiv-1 infection. Muscle Nerve.

[6] Mawuntu A.H., Mahama C.N., Khosama H., Estiasari R., Imran D. (2018). Early detection of peripheral neuropathy using stimulated skin wrinkling test in human immunodeficiency virus infected patients: A cross-sectional study. Medicine.

[7] Dalakas M.C. (2001). Peripheral neuropathy and antiretroviral drugs. J. Peripher. Nerv. Syst..

[8] Merlin J.S., Westfall A.O., Heath S.L., Goodin B.R., Stewart J.C., Sorge R.E., Younger J. (2017). Brief report: Il-1β levels are associated with chronic multisite pain in people living with hiv. Am. J. Ther..

[9] Orecchioni M., Ghosheh Y., Pramod A.B., Ley K. (2019). Macrophage polarization: Different gene signatures in m1(lps+) vs. Classically and m2(lps-) vs. Alternatively activated macrophages. Front. Immunol..

[10] Sica A., Mantovani A. (2012). Macrophage plasticity and polarization: In vivo veritas. J. Clin. Investig..

[11] Cabot P.J., Carter L., Gaiddon C., Zhang Q., Schäfer M., Loeffler J.P., Stein C. (1997). Immune cell-derived beta-endorphin. Production, release, and control of inflammatory pain in rats. J. Clin. Investig..

[12] Cabot P.J., Carter L., Schäfer M., Stein C. (2001). Methionine-enkephalin-and dynorphin a-release from immune cells and control of inflammatory pain. Pain.

[13] Mousa S.A., Shakibaei M., Sitte N., Schäfer M., Stein C. (2004). Subcellular pathways of beta-endorphin synthesis, processing, and release from immunocytes in inflammatory pain. Endocrinology.

[14] Sibinga N.E.S., Goldstein A. (1988). Opioid peptides and opioid receptors in cells of the immune system. Annu. Rev. Immunol..

[15] Pannell M., Labuz D., Celik M., Keye J., Batra A., Siegmund B., Machelska H. (2016). Adoptive transfer of m2 macrophages reduces neuropathic pain via opioid peptides. J. Neuroinflamm..

[16] Aggarwal S., DeBerry J.J., Ahmad I., Lynn P., Dewitte C., Malik S., Merlin J.S., Goodin B.R., Heath S.L., Matalon S. (2020). Heme attenuates beta-endorphin levels in leukocytes of hiv positive individuals with chronic widespread pain. Redox Biol..

[17] Fortes G.B., Alves L.S., De Oliveira R., Dutra F.F., Rodrigues D., Fernandez P.L., Souto-Padron T., DE Rosa M.J., Kelliher M., Golenbock D. (2012). Heme induces programmed necrosis on macrophages through autocrine tnf and ros production. Blood.

[18] Vinchi F., Costa da Silva M., Ingoglia G., Petrillo S., Brinkman N., Zuercher A., Cerwenka A., Tolosano E., Muckenthaler M.U. (2016). Hemopexin therapy reverts heme-induced proinflammatory phenotypic switching of macrophages in a mouse model of sickle cell disease. Blood.

[19] Aggarwal S., Lam A., Bolisetty S., Carlisle M.A., Traylor A., Agarwal A., Matalon S. (2016). Heme attenuation ameliorates irritant gas inhalation-induced acute lung injury. Antioxid. Redox Signal..

[20] Belcher J.D., Chen C., Nguyen J., Milbauer L., Abdulla F., Alayash A.I., Smith A., Nath K.A., Hebbel R.P., Vercellotti G.M. (2014). Heme triggers tlr4 signaling leading to endothelial cell activation and vaso-occlusion in murine sickle cell disease. Blood.

[21] Figueiredo R.T., Fernandez P.L., Mourao-Sa D.S., Porto B.N., Dutra F.F., Alves L.S., Oliveira M.F., Oliveira P.L., Graça-Souza A.V., Bozza M.T. (2007). Characterization of heme as activator of toll-like receptor 4. J. Biol. Chem..

[22] Dutra F.F., Alves L.S., Rodrigues D., Fernandez P.L., de Oliveira R.B., Golenbock D.T., Zamboni D.S., Bozza M.T. (2014). Hemolysis-induced lethality involves inflammasome activation by heme. Proc. Natl. Acad. Sci. USA.

[23] Moin A.S.M., Sathyapalan T., Diboun I., Atkin S.L., Butler A.E. (2021). Identification of macrophage activation-related biomarkers in obese type 2 diabetes that may be indicative of enhanced respiratory risk in covid-19. Sci. Rep..

[24] Matsuda M., Huh Y., Ji R.-R. (2018). Roles of inflammation, neurogenic inflammation, and neuroinflammation in pain. J. Anesthesia.

[25] Grassin-Delyle S., Abrial C., Salvator H., Brollo M., Naline E., Devillier P. (2020). The role of toll-like receptors in the production of cytokines by human lung macrophages. J. Innate Immun..

[26] Nicotra L., Loram L.C., Watkins L.R., Hutchinson M.R. (2012). Toll-like receptors in chronic pain. Exp. Neurol..

[27] Bruno K., Woller S., Miller Y.I., Yaksh T.L., Wallace M., Beaton G., Chakravarthy K. (2018). Targeting toll-like receptor-4 (tlr4)-an emerging therapeutic target for persistent pain states. Pain.

[28] Genin M., Clement F., Fattaccioli A., Raes M., Michiels C. (2015). M1 and m2 macrophages derived from thp-1 cells differentially modulate the response of cancer cells to etoposide. BMC Cancer.

[29] Chanput W., Mes J.J., Savelkoul H.F.J., Wichers H.J. (2013). Characterization of polarized thp-1 macrophages and polarizing ability of lps and food compounds. Food Funct..

[30] Kegel V., Deharde D., Pfeiffer E., Zeilinger K., Seehofer D., Damm G. (2016). Protocol for isolation of primary human hepatocytes and corresponding major populations of non-parenchymal liver cells. J. Vis. Exp..

[31] Chatterjee T., Pattanayak R., Ukil A., Chowdhury S., Bhattacharyya M. (2019). Autophagy protects peripheral blood mononuclear cells against inflammation, oxidative and nitrosative stress in diabetic dyslipidemia. Free. Radic. Biol. Med..

[32] Chatterjee T., De D., Chowdhury S., Bhattacharyya M. (2020). Nuclear factor nf-κb1 functional promoter polymorphism and its expression conferring the risk of type 2 diabetes-associated dyslipidemia. Mamm. Genome.

[33] Bonin R.P., Bories C., De Koninck Y. (2014). A simplified up-down method (sudo) for measuring mechanical nociception in rodents using von frey filaments. Mol. Pain.

[34] CChoi J., Liu R.-M., Kundu R.K., Sangiorgi F., Wu W., Maxson R., Forman H.J. (2000). Molecular mechanism of decreased glutathione content in human immunodeficiency virus type 1 tat-transgenic mice. J. Biol. Chem..

[35] Staal F.J.T., Roederer M., Israelski D.M., Bubp J., Mole L.A., McSHANE D., Deresinski S.C., Ross W., Sussman H., Raju P.A. (1992). Intracellular glutathione levels in t cell subsets decrease in hiv-infected individuals. AIDS Res. Hum. Retroviruses.

[36] Kline E.R., Kleinhenz D.J., Liang B., Dikalov S., Guidot D.M., Hart C.M., Jones D.P., Sutliff R.L. (2008). “Vascular oxidative stress and nitric oxide depletion in hiv-1 transgenic rats are reversed by glutathione restoration”. Am. J. Physiol. Heart Circ. Physiol..

[37] East J., Blanton L.S. (2011). Symptomatic hyperbilirubinemia secondary to dapsone-induced hemolysis and atazanavir therapy. Antimicrob. Agents Chemother..

[38] Camara-Lemarroy C.R., Flores-Cantu H., Calderon-Hernandez H.J., A Diaz-Torres M., Villareal-Velazquez H.J. (2015). Drug-induced haemolysis, renal failure, thrombocytopenia and lactic acidosis in patients with hiv and cryptococcal meningitis: A diagnostic challenge. Int. J. STD AIDS.

[39] Rodríguez-Nóvoa S., Morello J., González M., Vispo E., Barreiro P., González-Pardo G., Jiménez-Nácher I., Gonzalez-Lahoz J., Soriano V. (2008). Increase in serum bilirubin in hiv/hepatitis-c virus-coinfected patients on atazanavir therapy following initiation of pegylated-interferon and ribavirin. Aids.

[40] Becker S., Fusco G., Fusco J., Balu R., Gangjee S., Brennan C., Feinberg J. (2004). Hiv-associated thrombotic microangiopathy in the era of highly active antiretroviral therapy: An observational study. Clin. Infect. Dis..

[41] Louw S., Gounden R., Mayne E.S. (2018). Gounden and E. S. Mayne. Thrombotic thrombocytopenic purpura (ttp)-like syndrome in the hiv era. Thromb. J..

[42] Burke P.A., Aljadir D., Raman T. (2010). Diagnosis, management, and pathogenesis of ttp/hus in an hiv positive patient. Del. Med J..

[43] Serpa J.A., Villarreal-Williams E., Giordano T.P. (2010). Prevalence of g6pd deficiency in a large cohort of hiv-infected patients. J. Infect..

[44] Spinazzola F., Barletta C., DeMartino G., Martini F., Natili S., Noto P., Ferri F., Tossini G., Visco G. (1995). Beta-endorphins acth and cortisol in csf and plasma of hiv infected patients. Riv. Eur. Sci. Med. Farmacol.

[45] Sandler N.G., Wand H., Roque A., Law M., Nason M.C., Nixon D.E., Pedersen C., Ruxrungtham K., Lewin S.R., Emery S. (2011). Plasma levels of soluble cd14 independently predict mortality in hiv infection. J. Infect. Dis..

[46] Celik M., Labuz D., Keye J., Glauben R., Machelska H. (2020). Il-4 induces m2 macrophages to produce sustained analgesia via opioids. J. Clin. Investig..

[47] Adisa O.A., Hu Y., Ghosh S., Aryee D., Osunkwo I., Ofori-Acquah S.F. (2013). Association between plasma free haem and incidence of vaso-occlusive episodes and acute chest syndrome in children with sickle cell disease. Br. J. Haematol..

[48] Ghosh S., Adisa O.A., Chappa P., Tan F., Jackson K.A., Archer D.R., Ofori-Acquah S.F. (2013). Extracellular hemin crisis triggers acute chest syndrome in sickle mice. J. Clin. Investig..

[49] Atri C., Guerfali F.Z., Laouini D. (2018). Role of human macrophage polarization in inflammation during infectious diseases. Int. J. Mol. Sci..

[50] Kapitzke D., Vetter I., Cabot P.J. (2005). Endogenous opioid analgesia in peripheral tissues and the clinical implications for pain control. Ther. Clin. Risk Manag..

[51] Wen S., Jiang Y., Liang S., Cheng Z., Zhu X., Guo Q. (2022). Opioids regulate the immune system: Focusing on macrophages and their organelles. Front. Pharmacol..

[52] Ma S.-F., Chen Y.-J., Zhang J.-X., Shen L., Wang R., Zhou J.-S., Hu J.-G., Lü H.-Z. (2015). Adoptive transfer of M2 macrophages promotes locomotor recovery in adult rats after spinal cord injury. Brain Behav. Immun..

[53] Willemen H.L., Eijkelkamp N., Carbajal A.G., Wang H., Mack M., Zijlstra J., Heijnen C.J., Kavelaars A. (2014). Monocytes/macrophages control resolution of transient inflammatory pain. J. Pain.

[54] Mosser D.M., Edwards J.P. (2008). Exploring the full spectrum of macrophage activation. Nat. Rev. Immunol..

[55] Martinez F.O., Gordon S. (2014). The m1 and m2 paradigm of macrophage activation: Time for reassessment. F1000Prime Rep..

[56] Jablonski K.A., Amici S.A., Webb L.M., Ruiz-Rosado J.D.D., Popovich P.G., Partida-Sanchez S., Guerau-De-Arellano M. (2015). Novel markers to delineate murine m1 and m2 macrophages. PLOS ONE.

[57] Chen W., Liu J., Meng J., Lu C., Li X., Wang E., Shan F. (2012). Macrophage polarization induced by neuropeptide methionine enkephalin (menk) promotes tumoricidal responses. Cancer Immunol. Immunother..

[58] Tedesco S., Bolego C., Toniolo A., Nassi A., Fadini G.P., Locati M., Cignarella A. (2015). Phenotypic activation and pharmacological outcomes of spontaneously differentiated human monocyte-derived macrophages. Immunobiology.

[59] Wang S., Zhang J., Sui L., Xu H., Piao Q., Liu Y., Qu X., Sun Y., Song L., Li D. (2017). Antibiotics induce polarization of pleural macrophages to m2-like phenotype in patients with tuberculous pleuritis. Sci. Rep..

[60] Dong H., Yang Y., Gao C., Sun H., Wang H., Hong C., Wang J., Gong F., Gao X. (2020). Lactoferrin-containing immunocomplex mediates antitumor effects by resetting tumor-associated macrophages to m1 phenotype. J. Immunother. Cancer.

[61] Trombetta A.C., Soldano S., Contini P., Tomatis V., Ruaro B., Paolino S., Brizzolara R., Montagna P., Sulli A., Pizzorni C. (2018). A circulating cell population showing both m1 and m2 monocyte/macrophage surface markers characterizes systemic sclerosis patients with lung involvement. Respir. Res..

[62] Ambarus C., Krausz S., van Eijk M., Hamann J., Radstake T., Reedquist K., Tak P., Baeten D. (2012). Systematic validation of specific phenotypic markers for in vitro polarized human macrophages. J. Immunol. Methods.

[63] Mily A., Kalsum S., Loreti M.G., Rekha R.S., Muvva J.R., Lourda M., Brighenti S. (2020). Polarization of m1 and m2 human monocyte-derived cells and analysis with flow cytometry upon mycobacterium tuberculosis infection. J. Vis. Exp..

[64] Zhou Y., Yoshida S., Kubo Y., Yoshimura T., Kobayashi Y., Nakama T., Yamaguchi M., Ishikawa K., Oshima Y., Ishibashi T. (2017). Different distributions of m1 and m2 macrophages in a mouse model of laser-induced choroidal neovascularization. Mol. Med. Rep..

[65] Duque G.A., Descoteaux A. (2014). Macrophage cytokines: Involvement in immunity and infectious diseases. Front. Immunol..

[66] Merle N.S., Grunenwald A., Figueres M.-L., Chauvet S., Daugan M., Knockaert S., Robe-Rybkine T., Noé R., May O., Frimat M. (2018). Characterization of renal injury and inflammation in an experimental model of intravascular hemolysis. Front. Immunol..

[67] Sindrilaru A., Peters T., Wieschalka S., Baican C., Baican A., Peter H., Hainzl A., Schatz S., Qi Y., Schlecht A. (2011). An unrestrained proinflammatory m1 macrophage population induced by iron impairs wound healing in humans and mice. J. Clin. Investig..

[68] HHaldar M., Kohyama M., So A.Y., Kc W., Wu X., Briseno C.G., Satpathy A.T., Kretzer N.M., Arase H., Rajasekaran N.S. (2014). Heme-mediated spi-c induction promotes monocyte differentiation into iron-recycling macrophages. Cell.

[69] Li M., Hou Q., Zhong L., Zhao Y., Fu X. (2021). Macrophage related chronic inflammation in non-healing wounds. Front. Immunol..

[70] Nieuwenhuizen L., Schutgens R.E., Coeleveld K., Mastbergen S.C., Roosendaal G., Biesma D.H., Lafeber F.P. (2014). Hemarthrosis in hemophilic mice results in alterations in m1-m2 monocyte/macrophage polarization. Thromb. Res..

[71] Lin T., Kwak Y.H., Sammy F., He P., Thundivalappil S., Sun G., Chao W., Warren H.S. (2010). Synergistic inflammation is induced by blood degradation products with microbial toll-like receptor agonists and is blocked by hemopexin. J. Infect. Dis..

[72] Guo L.H., Schluesener H.J. (2007). The innate immunity of the central nervous system in chronic pain: The role of toll-like receptors. Cell. Mol. Life Sci..

[73] Christianson C.A., Dumlao D.S., Stokes J.A., Dennis E.A., Svensson C.I., Corr M., Yaksh T.L. (2011). Spinal tlr4 mediates the transition to a persistent mechanical hypersensitivity after the resolution of inflammation in serum-transferred arthritis. Pain.

[74] Sorge R.E., LaCroix-Fralish M.L., Tuttle A.H., Sotocinal S.G., Austin J.-S., Ritchie J., Chanda M.L., Graham A.C., Topham L., Beggs S. (2011). Spinal cord toll-like receptor 4 mediates inflammatory and neuropathic hypersensitivity in male but not female mice. J. Neurosci..

[75] Kim D., Kim M.A., Cho I.-H., Kim M.S., Lee S., Jo E.-K., Choi S.-Y., Park K., Kim J.S., Akira S. (2007). A critical role of toll-like receptor 2 in nerve injury-induced spinal cord glial cell activation and pain hypersensitivity. J. Biol. Chem..

[76] Qian N.-S., Liao Y.-H., Feng Q.-X., Tang Y., Dou K.-F., Tao K.-S. (2011). Spinal toll like receptor 3 is involved in chronic pancreatitis-induced mechanical allodynia of rat. Mol. Pain.

[77] Das N., Dewan V., Grace P.M., Gunn R.J., Tamura R., Tzarum N., Watkins L.R., Wilson I.A., Yin H. (2016). Hmgb1 activates proinflammatory signaling via tlr5 leading to allodynia. Cell Rep..

[78] Park C.-K., Xu Z.-Z., Berta T., Han Q., Chen G., Liu X.-J., Ji R.-R. (2014). Extracellular micrornas activate nociceptor neurons to elicit pain via tlr7 and trpa1. Neuron.

[79] David B.T., Ratnayake A., Amarante M.A., Reddy N.P., Dong W., Sampath S., Heary R.F., Elkabes S. (2013). A toll-like receptor 9 antagonist reduces pain hypersensitivity and the inflammatory response in spinal cord injury. Neurobiol. Dis..

[80] Stokes J.A., Corr M., Yaksh T.L. (2013). Spinal toll-like receptor signaling and nociceptive processing: Regulatory balance between tirap and trif cascades mediated by tnf and ifnβ. Pain.

[81] Chang J.J., Lacas A., Lindsay R.J., Doyle E., Axten K.L., Pereyra F., Rosenberg E.S., Walker B.D., Allen T., Altfeld M. (2012). Differential regulation of toll-like receptor pathways in acute and chronic hiv-1 infection. Aids.

[82] Hernández J.C., Stevenson M., Latz E., Urcuqui-Inchima S. (2012). Hiv type 1 infection up-regulates tlr2 and tlr4 expression and function in vivo and in vitro. Hum. Retroviruses.

[83] Min H., Choi B., Jang Y.H., Cho I.-H., Lee S.J. (2017). Heme molecule functions as an endogenous agonist of astrocyte tlr2 to contribute to secondary brain damage after intracerebral hemorrhage. Mol. Brain.

[84] Del Cornò M., Cappon A., Donninelli G., Varano B., Marra F., Gessani S. (2016). Hiv-1 gp120 signaling through tlr4 modulates innate immune activation in human macrophages and the biology of hepatic stellate cells. J. Leukoc. Biol..

[85] Ben Haij N., Leghmari K., Planès R., Thieblemont N., Bahraoui E. (2013). Hiv-1 tat protein binds to tlr4-md2 and signals to induce tnf-alpha and il-10. Retrovirology.

