# Supplementary material for: Heme-Induced Macrophage Phenotype Switching and Impaired Endogenous Opioid Homeostasis Correlate with Chronic Widespread Pain in HIV
Source: Cells. 2023 Jun 6;12(12):1565. doi: 10.3390/cells12121565 (PMC10297192; doi:10.3390/cells12121565)
Supplement: Supplementary file 1 [file cells-12-01565-s001.zip › cells-2403261-supplementary.pdf]

Supplementary Table S1

Antibody	Source	Clone	Catalog Number
Brilliant Violet 605 anti-human CD11b	BioLegend	ICRF44	301332
PE/Cyanine7 anti-human CD163	BioLegend	GHI/61	333614
Brilliant Violet 421 anti-human CD206 (MMR)	BioLegend	15-2	321126
APC anti-human CD80	BioLegend	2D10	305220
PE/Dazzle 594 anti-human CD32	BioLegend	FUN-2	303218
Brilliant Violet 711 anti-human CD86	BioLegend	IT2.2	305440
PE/Fire 700 anti-human CD38	BioLegend	S17015A	397121
APC anti-mouse F4/80 Antibody	BioLegend	BM8	123116
PE anti-mouse CD86 Antibody	BioLegend	GL-1	105008
Brilliant Violet 421 anti-mouse CD163 Antibody	BioLegend	S15049I	155309
Brilliant Violet 510 anti-mouse CD80 Antibody	BioLegend	16-10A1	104741
Alexa Fluor 488 anti-mouse/human CD11b Antibody	BioLegend	M1/70	101217
PE/Cyanine7 anti-mouse CD206 (MMR) Antibody	BioLegend	C068C2	141720
7AAD viability staining solution	BioLegend	N/A	420403
TruStain FcX™ PLUS (anti-mouse CD16/32) Antibody	BioLegend	N/A	156603

Supplementary Table S1. The table shows the source of all antibodies that were used to perform FACS analysis in the study.

Gene	Forward Primer	Reverse Primer
IL-6	TACCACTTCACAAGTCGGAGGC	CTGCAAGTGCATCATCGTTGTTC
TNF-α	GGTGCCTATGTCTCAGCCTCTT	GCCATAGAACTGATGAGAGGGAG
HO 1	CACTCTGGAGATGACACCTGAG	GTGTTCCTCTGTCAGCATCACC
Arg1	CATTGGCTTGCGAGACGTAGAC	GCTGAAGGTCTCTTCCATCACC
YM1	TACTCACTTCCACAGGAGCAGG	CTCCAGTGTAGCCATCCTTAGG
IL-1B	TGGACCTTCCAGGATGAGGACA	GTTCATCTCGGAGCCTGTAGTG
IL-10	CGGGAAGACAATAACTGCACCC	CGGTTAGCAGTATGTTGTCCAGC
Fpn or SLc40a1	CCATAGTCTCTGTCAGCCTGCT	CTTGCAGCAACTGTGTCACCGT
Erg 2	CCTTTGACCAGATGAACGGAGTG	CTGGTTTCTAGGTGCAGAGATGG
ACTB	CATTGCTGACAGGATGCAGAAGG	TGCTGGAAGGTGGACAGTGAGG
GAPDH	CATCACTGCCACCCAGAAGACTG	ATGCCAGTGAGCTTCCCGTTCAG

Supplementary Table S2

Mice Primers

Primers for THP-1 cells

Gene	Forward Primer	Reverse Primer
IL-10	TCTCCGAGATGCCTTCAGCAGA	TCAGACAAGGCTTGGCAACCCA
TNF alpha	CTCTTCTGCCTGCTGCACTTTG	ATGGGCTACAGGCTTGTCACTC
Cox 2	CGGTGAAACTCTGGCTAGACAG	GCAAACCGTAGATGCTCAGGGA
APOL3	CCAGTGCATCTGCAAATCCTGC	CTTTCTGCTGCACATATTCGTCC
IRF1	GAGGAGGTGAAAGACCAGAGCA	TAGCATCTCGGCTGGACTTCGA
IL-6	AGACAGCCACTCACCTCTTCAG	TTCTGCCAGTGCCTCTTTGCTG
Beta actin (ACTB)	CACCATTGGCAATGAGCGGTTC	AGGTCTTTGCGGATGTCCACGT
GAPDH	GTCTCCTCTGACTTCAACAGCG	ACCACCCTGTTGCTGTAGCCAA
IL-1 Beta	CCACAGACCTTCCAGGAGAATG	GTGCAGTTCAGTGATCGTACAGG
IRF5	TATGCCATCCGCCTGTGTCAGT	GCCCTTTTGGAACAGGATGAGC
Arg 1	TCATCTGGGTGGATGCTCACAC	GAGAATCCTGGCACATCGGGAA
CHI3L1 (Ym1)	AGGTCACCATTGACAGCAGC	ATCCTCCTGACCTCGGAACA
Macrophage Inflammatory Protein 4 (CCL18)	GTTGACTATTCTGAAACCAGCCC	GTCGCTGATGTATTTCTGGACCC

Supplementary Table S2. The table shows all the primers that were used to perform real time PCR in the study.

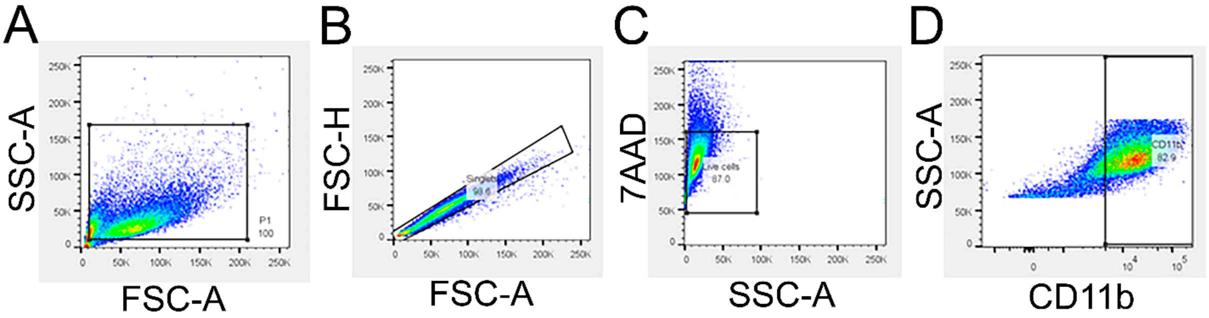

Supplementary Figure S1. THP-1 macrophages gating strategy. Representative dot plot shows Forward scatter (FSC) and side scatter (SSC) properties of PMA stimulated THP-1 macrophages (A). The FSC-A/FSC-H plot shows manual gating of single cells proportionate to area and height (B). Next, the dot plot shows live cell gating using 7AAD (viability dye) (C). After the selection of single live cells, the analysis of polarization markers was performed on CD11b positive cells (D).

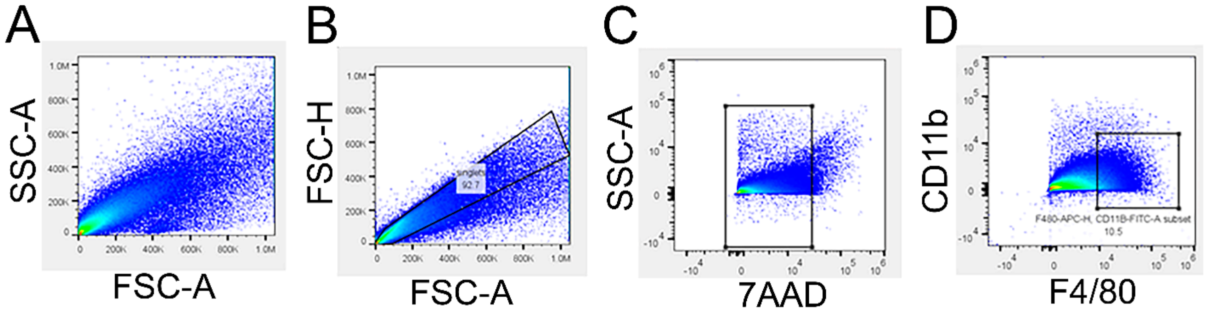

Supplementary Figure S2. Liver cells gating strategy. Representative dot plot shows Forward scatter (FSC) and side scatter (SSC) properties of liver macrophage (A). The FSC-H/FSC-A plot shows manual gating of single cells proportionate for area and height (B). Next, the dot plot shows live cell gating using 7AAD (viability dye) (C). After the selection of single live cells, the analysis of polarization markers was performed on all F4/80 positive cells with low CD11b expression (D).
